# *Vernonia amygdalina* Leaf Extract Induces Apoptosis in HeLa Cells: A Metabolomics and Proteomics Study

**DOI:** 10.3390/ph17081079

**Published:** 2024-08-16

**Authors:** Pawitrabhorn Samutrtai, Yodying Yingchutrakul, Kriangsak Faikhruea, Tirayut Vilaivan, Vorrapon Chaikeeratisak, Jaruwan Chatwichien, Sucheewin Krobthong, Chanat Aonbangkhen

**Affiliations:** 1 Department of Pharmaceutical Sciences, Faculty of Pharmacy, Chiang Mai University, Chiang Mai 50200, Thailand; 2 National Center for Genetic Engineering and Biotechnology, National Science and Technology Development Agency (NSTDA), Pathum Thani 12120, Thailand; yodying.yin@biotec.or.th; 3 Organic Synthesis Research Unit (OSRU), Department of Chemistry, Faculty of Science, Chulalongkorn University, Bangkok 10330, Thailand; k.faikhruea@gmail.com (K.F.); vtirayut@chula.ac.th (T.V.); 4 Department of Biochemistry, Faculty of Science, Chulalongkorn University, Bangkok 10330, Thailand; vorrapon.c@chula.ac.th; 5 Program in Chemical Sciences, Chulabhorn Graduate Institute (CGI), Bangkok 10210, Thailand; jaruwanc@cgi.ac.th; 6 Center of Excellence in Natural Products Chemistry (CENP), Department of Chemistry, Faculty of Science, Chulalongkorn University, Bangkok 10330, Thailand; sucheewin82@gmail.com (S.K.); chanat.a@chula.ac.th (C.A.); 7 Center of Excellence on Petrochemical and Materials Technology, Chulalongkorn University, Bangkok 10330, Thailand

**Keywords:** medicinal plants, metabolomics, proteomics, cancer, apoptosis, JNK, p53, caspase-9, PTEN, XBP1

## Abstract

Medicinal plants produce various bioactive molecules with potential anti-cancer properties with favorable safety profiles. We aimed to investigate the comprehensive composition of *Vernonia amygdalina* leaf extract and its cytotoxic effects via apoptosis in HeLa cells. The metabolomics approach using LC-MS/MS was conducted to gather the metabolite profile of the extract. Proteomics was performed to understand the comprehensive mechanistic pathways of action. The apoptosis was visualized by cellular staining and the apoptotic proteins were evaluated. *V. amygdalina* leaf extract exhibited dose-dependent cytotoxic effects on both HeLa and Vero cells after 24 h of exposure in the MTT assay with the IC_50_ values of 0.767 ± 0.0334 and 4.043 ± 0.469 µg mL^−1^, respectively, which demonstrated a higher concentration required for Vero cell cytotoxicity. The metabolomic profile of 112 known metabolites specified that the majority of them were alkaloids, phenolic compounds, and steroids. Among these metabolites, deacetylvindoline and licochalcone B were suggested to implicate cytotoxicity. The cytotoxic pathways involved the response to stress and cell death which was similar to doxorubicin. The upstream regulatory proteins, phosphatase and tensin homolog deleted on chromosome ten (PTEN) and X-box binding protein 1 (XBP1), were significantly altered, supporting the regulation of apoptosis and cell death. The levels of apoptotic proteins, c-Jun N-terminal kinases (JNK), p53, and caspase-9 were significantly increased. The novel insights gained from the metabolomic profiling and proteomic pathway analysis of *V. amygdalina* leaf extract have identified crucial components related to apoptosis induction, highlighting its potential to develop future chemotherapy.

## 1. Introduction

Cancer is a complex disease with a broad group of disorders. It has a major impact on the human population and complicates the healthcare industry and public policy. In 2022, there were 9.7 million people died from cancer, with nearly 20 million new cases reported worldwide [1]. This has led to the extensive screening of synthetic and natural compounds, intending to develop new chemotherapeutic treatments that are more effective, selective, and less hazardous. Medicinal plants contain a wide array of bioactive molecules with potential anti-cancer properties, of which some were approved by the Food and Drug Administration (FDA) as chemotherapy. The prominent examples of plant-derived anti-cancer compounds are vincristine, vinblastine, and taxanes, which are secondary metabolites with effectiveness in combating cancer cells by targeting the cellular cytoskeleton [2].

Apoptosis is a controlled process that helps eliminate unwanted cells via programed cell death, which is crucial for tissue homeostasis. Abnormalities in apoptosis can lead to diseases like cancer and autoimmune disorders. Cancer is caused by mutations in tumor suppressors or oncogenes, leading to resistance against apoptosis and requiring higher drug doses for treatment [3]. Inducing apoptosis in malignant cells plays a crucial role in cancer therapy, however, the nonspecific initiation of apoptosis may be harmful to normal cells as well. Therefore, understanding apoptotic pathways and developing pro-apoptotic agents with negligible side effects are essential for novel cancer treatments and apoptosis resistance [4]. There have been reports of plant-derived compounds with potential pro-apoptotic activities. Terpenoids such as ursolic acid, isolated from different herbs like rosemary, oregano, lavender, and thyme, were shown to inhibit breast cancer stem cells by activating Akt [5]. Betulinic acid from the evergreen plant of the Rhamnaceae family upregulated Bax, and inhibited Bcl-2, thereby activating caspase-9 and downstream caspase-3, -7 leading to the apoptosis in leukemia stem cells [6]. Polyphenols such as resveratrol from grapes, peanuts, and mulberry could also induce apoptosis of osteoma and glioma stem cells by activating the p53 gene [7]. Curcumin from the rhizome of *Curcuma longa* Linn. (Zingiberaceae) was shown to regulate the apoptotic pathway by blocking the Akt/FoxM1 pathway, thus exhibiting the anti-proliferative activity of gastric cancer stem cells [8]. Alkaloids are also among the natural compounds that were reported to induce apoptosis. Berberine isolated from Rhizoma coptidis has been shown to inhibit various tumors such as breast cancer, pancreatic cancer, lung cancer, and other tumors by regulating the Hedgehog pathway [9].

*Vernonia amygdalina*, or bitter leaf, is a small- to medium-sized shrub or tree belonging to the Asteraceae family native to tropical regions of Africa and Asia [10]. The leaf extract from *V. amygdalina* was reported to be composed of alkaloids, flavonoids, tannins and saponins, terpenes and terpenoids, steroids, and cardiac glycosides, making it renowned for its extensive pharmacological activities [11]. It exhibited significant antidiabetic effects, displayed strong antioxidant and anti-inflammatory properties, demonstrated antimalarial activity, and possessed broad-spectrum antimicrobial abilities against bacteria, fungi, and viruses [12]. In terms of anti-cancer properties, *V. amygdalina* extract was reported to be a potential cancer treatment. It was suggested to destroy cancer cells by inducing apoptosis in hepatoma via the inhibition of the PI3K/Akt signaling pathway [13] and exhibited cytotoxicity in prostate cancer cells by inhibiting cell growth, promoting cell cycle arrest and apoptosis through caspase-3 signaling cascade, and inducing DNA fragmentation [14]. It also displayed cardioprotective effects in rats when given concomitantly with the anti-cancer agent, doxorubicin [15]. Although the leaf extract of *V. amygdalina* was previously reported to demonstrate an apoptotic effect, information on the main components that caused cytotoxicity was still missing. 

The omics approach has been developed to understand the complexity of biological systems at different levels of biological molecules. This technology engages in screening and resolving a large amount of information, demonstrating the structural and functional background of a given entity using high-performance liquid chromatography paired with tandem mass spectrometry (LC-MS/MS), a powerful piece of analytical equipment that integrates the separating ability of liquid chromatography with the sensitive and selective mass analysis of two sequential mass analysis allowing the distinguished identification of compounds [16]. Metabolomics involves small molecules, commonly known as metabolites, whereas proteomics focuses on a set of proteins available within the biological system. Both metabolomics and proteomics play crucial roles in the discovery of new drugs from plants by providing comprehensive insights into the pharmacological actions of plant-derived substances. Metabolomics enables the detailed profiling of metabolites and the identification of bioactive compounds. Proteomics complements this by revealing interactions and changes in pathways which validates the pharmacological effects [17]. By integrating metabolomics and proteomics, we can understand a holistic view of how the particular substances from plants exert their effects, consequently accelerating the development of effective and safe therapeutic agents.

Although *V. amygdalina* leaf extract was reported to present apoptotic effects in various cells, the comparative safety profile to normal cells has not been verified. Moreover, the link between the composition of the extract and the comprehensive molecular pathways has not been established. In this study, we aimed to identify the composition of *V. amygdalina* leaf extract that possesses cytotoxic effects on the cancerous HeLa cells focusing on apoptotic activation. The study utilized the approaches of metabolomics to obtain the profile of substances in the extract that might be crucial for the cytotoxic effects, as well as proteomics for achieving comprehensive information on molecular mechanistic pathways and potential markers for the apoptosis induced by *V. amygdalina* extract. The metabolomic profiling using LC-MS/MS would provide the missing information on the main components of *V. amygdalina* leaf extract that influenced the cancer cytotoxicity, specifically the apoptotic pathways supported by the proteomic analysis and the quantification of apoptotic markers. The findings from this study would be fundamental for the further evaluation of the development of *V. amygdalina* leaf extract for cancer treatment.

## 2. Results and Discussion

### 2.1. Cell Cytotoxicity

**Figure 1:**
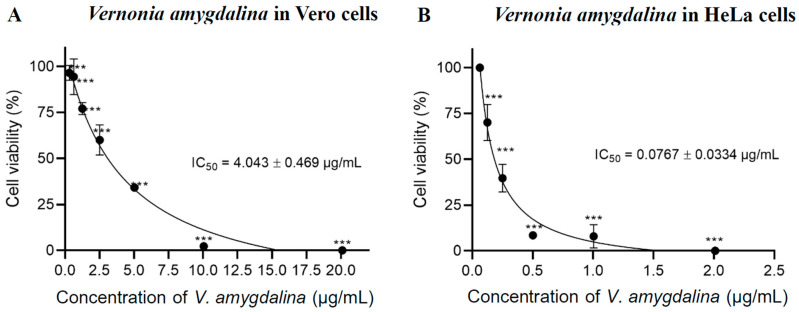
Cell viability and IC_50_ using MTT assay of *V. amygdalina* in (**A**) Vero cells and (**B**) HeLa cells, shown as the mean of three independent replicates ± S.D. (n = 3). Statistical significance was determined using one-way ANOVA and presented as *** *p* < 0.001 vs. control (0.1% DMSO).

The cytotoxicity effect of *V. amygdalina* leaf extract demonstrated cytotoxic effects on cancer cells in a dose-dependent manner as the cytotoxic concentration increased in both HeLa and Vero cells. However, the half-maximal inhibitory concentration (IC_50_) to kill Vero cells was higher than the cytotoxic concentration in HeLa cells after being treated for 24 h. The IC_50_ values of *V. amygdalina* leaf extract in Vero cells and HeLa cells were 4.043 ± 0.469 µg mL^−1^ (Figure 1A) and 0.0767 ± 0.0334 µg mL^−1^ (Figure 1B), respectively. These findings indicated that the *V. amygdalina* leaf extract exhibited selective cytotoxicity against cancer cells as it required a higher dose to kill normal cells. The leaf extract of *V. amygdalina* has been reported for its cytotoxic effects against cancer cells in various studies. Harefa and colleagues (2022) established the IC_50_ of ethyl acetate extract of *V. amygdalina* leaf in human hepatoma HepG2 cells to be 19.91 ± 0.24 µg/mL, however, its toxicity against normal cells was not demonstrated in this paper [18]. Nkono et al. (2022) also demonstrated the antiproliferative effects of aqueous alcoholic *V. amygdalina* leaf extract on human osteosarcoma (MG-63) cells at 125 and 250 μg mL^−1^, without cytotoxicity on rabbit primary dermal fibroblasts (RPDFs) at these concentrations [19].

### 2.2. The Metabolomics Profile of V. amygdalina Leaf Extract

**Figure 2:**
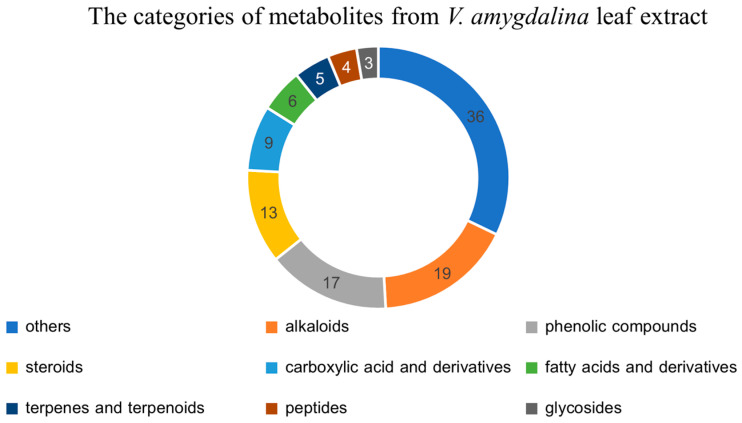
The metabolomics profile of the extract from *V. amygdalina* was categorized into 9 groups: alkaloids, phenolic compounds, steroids, carboxylic acid and derivatives, fatty acids and derivatives, terpenes and terpenoids, peptides, glycosides, and others. The most identified metabolites were alkaloids, phenolic compounds, and steroids.

The LC-MS/MS identified 112 known metabolites from the leaf extract of *V. amygdalina*. These metabolites were categorized based on their chemical structures into alkaloids, phenolic compounds, steroids, carboxylic acid and derivatives, fatty acids and derivatives, terpenes and terpenoids, peptides, glycosides, and others. The metabolomic profile of *V. amygdalina* leaf extract is depicted as a pie chart in Figure 2. The top three most abundant categories of metabolites were alkaloids with 19 identified substances, 17 phenolic compounds, and 13 steroids. Although there were 36 metabolites in the “others” category, they were not recognized as the major secondary metabolites in plants. Based on the phytochemical screening, it was also previously reported that alkaloids, tannins, flavonoids, saponins, triterpenoids, steroids, and glycosides were the major composition of the extract from the variation in *V. amygdalina* [11,20]. The FT-IR spectroscopy also identified the chemicals with functional groups OH, C-H, C=C, and C=O, indicating the presence of alkanes (C-H stretching), alkenes (C=C stretching), carboxylic acids (C=O stretching), and alcohols (OH stretching) in the methanol extract of *V. amygdalina* [21]. 

The majority of secondary metabolites found in *V. amygdalina* extract were alkaloids which were established as substances that induce apoptosis and some have been developed into current cancer treatment [2]. One of the most abundant alkaloids identified in the extract was deacetylvindoline, as shown in Table 1, and the MS2 spectrum in Supplementary Figure S2. Vindoline, an indole alkaloid, is a member of the Vinca alkaloids that was initially extracted from *Catharanthus roseus* and is a precursor for the synthesis of the chemotherapeutic drugs, vinblastine and vincristine [22,23]. The discovery of this alkaloid may explain the cytotoxic effect in cancer cells. However, other compositions in *V. amygdalina* extract may help mitigate the severity of cell death and may even exhibit a cytoprotective effect as the higher concentration of IC_50_ was required to kill normal cells. The cytoprotective substances that were established to mitigate oxidative stress and inflammation are flavonoids and phenolic compounds. Licochalcone B was one of the highest quantified phenolic compounds in *V. amygdalina* leaf extract. It is a bioactive chalcone with anti-inflammatory and antioxidative effects [24]. Moreover, licochalcone B was also reported to have a cytotoxic effect on hepatoma HepG2 cells via apoptotic activation [25]. Therefore, it was likely that these compounds from *V. amygdalina* leaf extract were involved with the apoptotic process.

In this study, we identified the various categories of metabolites and main components in *V. amygdalina* leaf extract using LC-MS/MS metabolomics analysis. The separating ability of high-performance liquid chromatography equipped with two sequential mass analyses provides powerful identification of plant-derived substances, and comprehensive plant metabolites were profiled efficiently [16].

### 2.3. Protein Pathway Analysis

**Figure 3:**
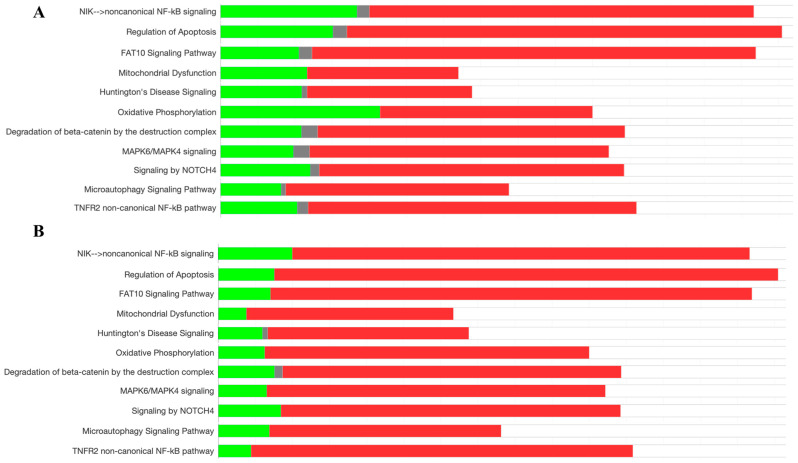
The percentage of upregulated (red), downregulated (green), and unchanged (gray) proteins functioning in the biological pathways of cell survival and response to stress shown as the comparison between HeLa cells exposed to *V. amygdalina* leaf extract and the non-treated control group (**A**), and HeLa cells exposed to doxorubicin compared with the control group (**B**).

The analysis of signaling pathways from the entire proteome of HeLa cells was performed using ingenuity pathway analysis (IPA). Differential proteome was compared between the *V. amygdalina* leaf extract exposed group versus the control non-treated group. Various pathways were identified to be activated and inhibited. The pathways involved in cell survival including the NF-κB pathway, the regulation of apoptosis, MAPK6/MAPK4 signaling, degradation of beta-catenin by the destruction complex, NOTCH4 signaling, and microautophagy signaling were triggered along with the pathways involved with the response to stress, i.e., the FAT10 signaling pathway, Huntington’s disease signaling, mitochondrial dysfunction, and oxidative phosphorylation as shown in Figure 3A. These patterns were also depicted in a higher intensity as the proteins working in these pathways were greatly regulated in HeLa cells treated with the anti-cancer drug, doxorubicin, compared with the control group as illustrated in Figure 3B. Doxorubicin is an anti-cancer therapy in which the concluding mechanism of killing tumors is apoptosis [26]. It could be suggested that the composition in the leaf extract of *V. amygdalina* activated the proteins functioning in apoptosis and stress response in a similar pattern as doxorubicin, although at a lower intensity.

**Figure 4:**
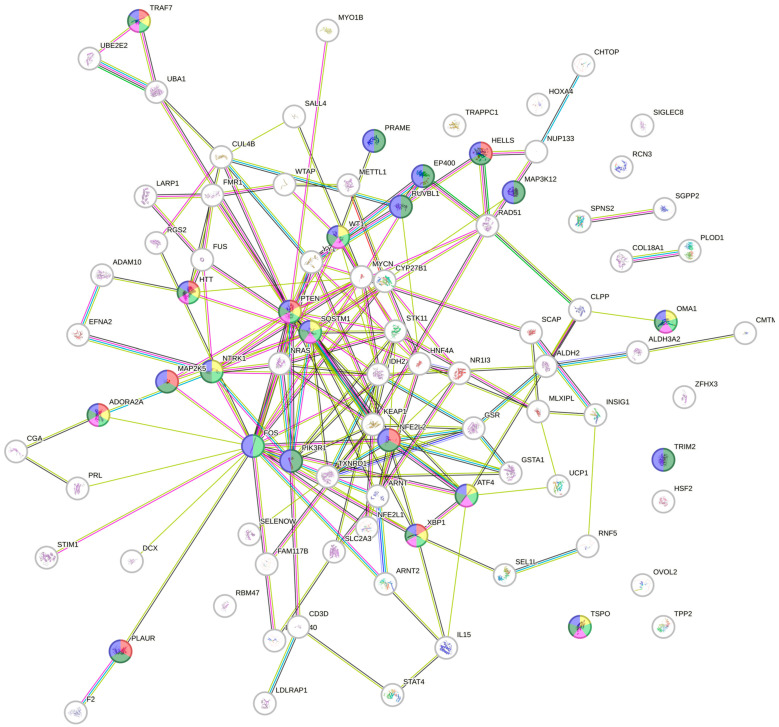
The protein–protein interaction map of the upstream regulators that significantly altered after the exposure to *V. amygdalina* leaf extract interaction prediction. The proteins functioning in GO biological processes are highlighted according to each process, i.e., GO:0042981 (regulation of apoptotic process) in dark green, GO:0010941 (regulation of cell death) in blue, GO:0010942 (positive regulation of cell death) in light green, GO:0043068 (positive regulation of programed cell death) in yellow, GO:2001233 (regulation of apoptotic signaling pathway) in red, and GO:0043065 (positive regulation of apoptotic process) in pink. The upstream protein regulators that were involved with all cell death and apoptosis pathways were PTEN, XBP1, and ADORA2A.

Regarding the prediction of upstream regulatory pathways in HeLa cells after the treatment with *V. amygdalina* leaf extract in HeLa cells, there were significant changes in 95 upstream regulators with *z*-scores ranging from −4.433 to +5.229 (the negative values referred to the inhibition, and the positive activation) with the top ten highest changed upstream regulators depicted in Table 2. All significant upstream regulators were input into STRING (https://cn.string-db.org/, accessed on 22 June 2024), an online protein interaction mapping database, to identify the connection between these regulators (Figure 4) and establish the potential gene ontology (GO) biological process. The GO biological processes involved with cell death and apoptosis were altered in the exposure to *V. amygdalina* leaf extract (Table 3) with the related upstream regulators highlighted in each pathway as illustrated in Figure 4. Among these pathways, we discovered that PTEN and XBP1 were crucial and functioned in all GO biological processes regarding cell death and apoptosis.

Phosphatase and tensin homolog deleted on chromosome ten (PTEN) is a phosphatidylinositol-3,4,5-triphosphate (PIP3) phospholipid phosphatase, an enzyme that primarily inhibits the PI3K/Akt pathway. This enzyme plays a vital role as a tumor suppressor and is often found to be mutated in a wide range of human cancers. It plays a significant role in regulating apoptosis through various mechanisms, such as antagonizing the PI3K/Akt signaling pathway, thus promoting apoptosis [27]. Additionally, PTEN regulates Ca^2+^ release from the endoplasmic reticulum (ER), influencing mitochondrial Ca^2+^ overload and inducing apoptosis [28]. Although the interaction between PTEN and X-box binding protein 1 (XBP1) was not specifically addressed, the role of XBP1 in regulating apoptosis was reported in the different molecular mechanisms. XBP1 was reported to regulate apoptosis by modulating the unfolded protein response (UPR) signaling pathway in response to endoplasmic reticulum (ER) stress [29]. However, XRP1 had a distinct effect on different cell types. XBP1 overexpression in β-cell impaired insulin secretion and enhanced apoptosis whereas it was reported to mitigate the ER stress-induced apoptosis in fibroblasts [30]. Therefore, the cytotoxic effects of *V. amygdalina* leaf extract might involve apoptosis via the activation of upstream regulators, PTEN and XBP1.

The proteomics approach using LC-MS/MS offers significant advantages in studying molecular pathways by providing insights into complex biological processes through the analysis of protein composition, which directly regulates cellular pathways. Tandem mass spectrometry enables the identification of thousands of proteins with high sensitivity and specificity, allowing for a comprehensive understanding of the pharmacological activities of plant-derived substances [17].

### 2.4. Apoptotic Cell Imaging and Apoptotic Protein Level Quantification

**Figure 5:**
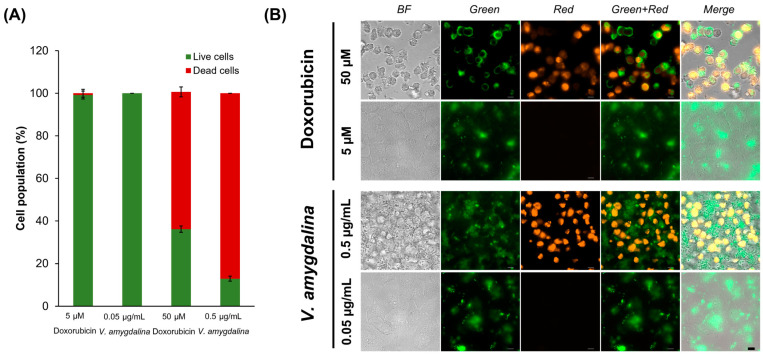
The intensity and images of apoptotic HeLa cells treated with 5 and 50 µM doxorubicin and *V. amygdalina* in the concentration of 0.05 and 0.5 µg/mL. (**A**): The intensity of fluorescence of dead and live cells, and (**B**): the images of green and red channels representing cells stained with annexin V and PI, respectively (Scale bar 10 µm).

The percentage of dead cells calculated by the fluorescence intensity from the image showed that 0.5 µg/mL concentration of *V. amygdalina* leaf extract induced apoptosis slightly better than in the 50 µM doxorubicin, while 5 µM doxorubicin and 0.05 µg/mL *V. amygdalina* extract did not induce the apoptosis in HeLa cells (Figure 5A). The green channel represents the detection of annexin V conjugated with the green fluorescent dye, FITC, the stain that interacts specifically with phosphatidyl serine, a target for the loss of plasma membrane asymmetry as presented in apoptosis. In addition, PI staining in the red channel indicated the dead cells as PI only crosses compromised membranes that lose integrity and bind with the DNA of those cells. We discovered the dead cells stained with both annexin V and PI, as depicted in Figure 5B.

**Figure 6:**
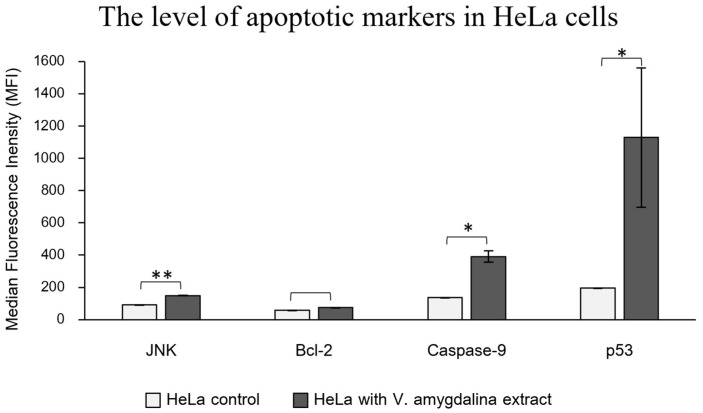
The levels of apoptotic markers, JNK, caspase-9, and p53 in the treatment of the *V. amygdalina* group (black bars), were significantly higher than in the control group (white bars) (** represents the *p*-value of 0.01 and * *p*-value of 0.05) whereas the level of anti-apoptotic protein, Bcl-2, increased insignificantly between two groups.

To verify the activation of apoptotic-related protein expression induced by *V. amygdalina* leaf extract, the immuno-based Luminex^®^ assay was performed to detect the apoptotic markers that are involved in the apoptotic signaling pathways. We detected significantly higher levels of three apoptotic proteins; JNK, caspase-9, and p53, in HeLa cells treated with the extract, while the increase in anti-apoptotic marker, Bcl-2 was insignificant, as shown in Figure 6. The molecular pathway involving JNK, p53, and caspase-9 in the process of apoptosis is intricate and crucial for cell fate determination. The c-Jun N-terminal kinases (JNKs) are part of the MAP kinases superfamily, which plays an essential role in controlling cellular development including cell growth, cell differentiation, and cell death [31]. The mediation of apoptosis by JNK relates to the phosphorylation of the p53 protein family, which consequently elevates the expression of Bax (Bcl2-associated X protein) and PUMA (p53 up-regulated modulator of apoptosis), the pro-apoptotic genes [32]. Moreover, JNKs have an important role in regulating apoptotic proteins in mitochondria [33]. The translocation of JNKs into mitochondria initiates apoptotic signaling by releasing cytochrome C from the mitochondrial inner membrane along with the apoptosomal secretion of Apaf-1, and caspase-9 activates the caspase-9 signaling pathway [34]. Since there was an established connection between the apoptotic proteins, JNK, p53, and caspase-9, which features a molecular cascade of apoptosis, it is suggested that the apoptosis could induce the cytotoxicity of HeLa cells treated with *V. amygdalina* extract.

The imaging of apoptotic cells and the levels of apoptotic proteins indicated the induction of apoptosis in HeLa cells exposed to *V. amygdalina* leaf extract. The fluorescent dyes represented the loss of plasma membrane asymmetry and integrity of apoptotic cells. The levels of pro-apoptotic proteins were significantly increased in HeLa cells treated with the leaf extract, while the level of anti-apoptotic protein was insignificant. These findings established the connection between *V. amygdalina* leaf extract and the cytotoxicity in cancer cells. 

From the metabolomics approach, we suggested that the main components in *V. amygdalina* leaf extract that related to the cytotoxicity via apoptosis were alkaloids and phenolic compounds. The composition of metabolites exhibited potential cytotoxic effects on cancerous HeLa cells at a lower concentration than that in the normal Vero cells. Proteins in the pathways of apoptosis and stress response were upregulated in a similar pattern to the group of doxorubicin, the anti-cancer treatment. The upstream regulator proteins in the regulation of apoptosis and cell death such as PTEN, XBP1, and ADORA2A were altered significantly. The verification of apoptosis using annexin V/PI staining along with the immuno-based Luminex^®^ assay confirmed the event of apoptosis in HeLa cells exposed to the leaf extract.

## 3. Materials and Methods

### 3.1. The Extraction of V. amygdalina Leaves

After being collected from the field, the *V. amygdalina* leaves were thoroughly cleaned and dried in an oven at 60 °C for 16 h. The dried leaves were ground into a coarse powder and subjected to maceration in hexane (analytical grade, Fisher, Hampton, NH, USA) to remove fatty compounds. This maceration process was stirred every 8 h for a total of 24 h. Following this, the hexane was filtered out using Whatman No.4 filter papers, leaving a coarse powder of *V. amygdalina* leaf. This powder was then extracted with ethyl acetate (EtOAc, Analytical grade, Fisher) by maceration and stirred intermittently every 8 h over 24 h, a process repeated three times. The combined extracts were filtered again using Whatman No.4 filter papers, and the EtOAc was removed with a rotary evaporator (Rotavapor^®^ R-300, Buchi, Flawil, Switzerland). The resulting dark green crude powder was stored in an amber glass bottle at 4 °C until it was used. The leaf extract was stored and used in experiments within three months.

### 3.2. Cell Cultures and Cytotoxicity Test

HeLa cells (CRM CCL-2™, ATCC, Manassas, VA, USA), which serve as a model for cancerous cells, and Vero cells (CRM CCL-81™, ATCC, Manassas, VA, USA), representing normal cells, were cultured in RPMI 1640 medium (Elabscience^®^, Houston, TX, USA) supplemented with 10% (*v*/*v*) fetal bovine serum (Gibco^®^, Waltham, MA, USA). The cells were maintained at 37 °C in a humidified incubator with 5% CO_2_ until they reached approximately 60–70% confluency in a T-75 cm² flask.

Cytotoxicity was assessed using the MTT assay according to an in-house protocol. Cell lines (1 × 10^4^ cells/mL) were plated in 96-well plates and incubated for 24 h at 37 °C in an atmosphere containing 5% CO_2_. The *V. amygdalina* leaf extract was tested at concentrations ranging from 0 to 2.5 µg mL^−1^ for HeLa cells and 0 to 20.0 µg mL^−1^ for Vero cells, with 0.1% DMSO in the culture medium, and incubated for 24 h. After removing the culture medium and test compounds, the cells were rinsed with phosphate-buffered saline (PBS, pH 7.4). A 5 mg mL^−1^ solution of 3-(4,5-dimethylthiazol-2-yl)-2,5-diphenyltetrazolium bromide (MTT) was then filled, followed by a 4 h incubation at 37 °C in 5% CO_2_. Absorbance readings were taken at 540 and 620 nm using a SpectraMax i3x multi-mode microplate reader. The percentage of cell viability was calculated using GraphPad Prism version 8.0, and statistical analysis was performed using one-way ANOVA (GraphPad, La Jolla, CA, USA). The experiment was conducted in triplicate.

### 3.3. The Metabolomics Profiling of V. amygdalina Leaf Extract

The profiling of metabolomics from *V. amygdalina* leaf extract was conducted using a Q-Exactive Quadrupole Orbitrap Mass Spectrometer in combination with an UltiMate 3000 LC system. A flow rate of 0.3 mL/min was used to introduce a 5 µL sample into the system. The Hypersil GOLD™ column (Thermo Scientific, Waltham, MA, USA) and the auto-sampler were maintained at temperatures of 60 °C and 6 °C, respectively. The mobile phase consisted of methanol/water (1:1) containing 0.1% formic acid (MP:A) and acetonitrile containing 0.1% formic acid (MP: B) (LC-MS grade, Sigma, Kawasaki-shi, Kanagawa, Japan). The gradient started with 99% MP:A and 5% MP: B for the first minute, then gradually increased to 95% MP: B over 23 min. The column was then washed with 100% MP: B for 5 min before returning to the initial conditions. After each injection, a blank sample (0.1% formic acid in methanol) was run. The mass spectrometer operated in positive mode with a spray voltage of 3.8 kV, using sheath gas and auxiliary gas flow rates of 48 and 11 arbitrary units (AU), respectively. The temperature at the capillary was conditioned to 350 °C. MS analysis was performed with dynamic exclusion and alternated between full MS scans and data-dependent MS/MS scans. Full MS scans covered a range of 75–700 *m*/*z* with a resolution of 120,000, an AGC target of 3e6, and a maximum injection time (IT) of 30 ms. MS/MS scans were performed at a resolution of 15,000, with an AGC target of 1e5 and a maximum IT of 50 ms, fragmenting the top five most intense ions. All data were processed using Xcalibur 3.1 software (Thermo Scientific, Waltham, MA, USA). LC-MS/MS conditions were optimized to achieve high resolution for separating overlapping peaks and capturing all plant metabolites in a sample, with a long scan time and broad scan range. Both LC and MS/MS parameters were tuned using multivariate analysis.

### 3.4. Sample Preparation for Label-Free Proteomics Analysis

HeLa cells in the treatment group were exposed to *V. amygdalina* leaf extract at a concentration of 0.1 µg mL^−1^ (IC_50_ from cytotoxicity test) for 24 h prior to protein extraction. The preparation of cell lysate followed an in-house protocol. After the collection, the cells were then lysed on an ice bath, in 200 µL of lysis buffer (0.2% Triton X-100, 2 mM TCEP, 5 mM NaCl, 10 mM HEPES-KOH, pH 8.0) containing a protease inhibitor cocktail with a probe tip sonication at a frequency of 20 kHz and 75% amplitude, with pulses of 2 s on and 3 s off, totaling 15 s. After a 24 h cold acetone precipitation (1:4 *v*/*v*), proteins were collected by centrifugation at 15,000× *g* for 10 min. The resulting protein pellet was dissolved in a solution containing 0.5% RapiGest SF (Waters, London, UK) and 5 mM NaCl in 15 mM ammonium bicarbonate. A total of 40 µg of protein underwent digestion. Disulfide bonds were reduced using 1 mM TCEP in 15 mM ammonium bicarbonate at 50 °C for 1 h, followed by the alkylation of sulfhydryl groups with 4 mM iodoacetamide (IAA) in 15 mM ammonium bicarbonate at room temperature for 45 min in the dark. The solution was purified using a desalting column (Zeba™ Spin Desalting Columns, 7K MWCO, 0.5 mL, ThermoFisher). The resulting flow-through was digested with trypsin (Promega, Walldorf, Germany) at an enzyme-to-protein ratio of 1:40 and incubated at 37 °C for 4 h. The tryptic peptides were then dried and immediately analyzed by MS/MS.

### 3.5. LC-MS/MS Settings and Configurations for Proteomics Analysis

Tryptic peptides were analyzed using an Orbitrap HF hybrid mass spectrometer coupled with an UltiMate 3000 LC system. Any residue salts in peptide samples were removed using a reverse-phase C18 PepMap 100 trapping column and the separation was performed by a C18 PepMapTM 100 capillary column. The solution of 0.1% formic acid was used to reconstitute dried peptides. The protonated peptides were introduced into the nanoLC system in the amount of 1.2 µg. The mobile phases were 0.1% formic acid in water (Phase A) and 95% acetonitrile with 0.1% formic acid (Phase B). Mass spectra were acquired in data-dependent acquisition mode, with full scans covering a mass range of 400–1600 *m*/*z*. The 15 most abundant peptide ions with charge states between 2 and 5 were subjected to fragmentation. Dynamic exclusion was conditioned for 18 s. Full scan mass spectra were recorded from *m*/*z* 400 to 1600, with an AGC target of 3 × 10^6^ ions and a resolution of 120,000. MS/MS scans were triggered once the AGC target reached 10^5^ ions, with dynamic exclusion applied for a 15 s window.

### 3.6. Biological Pathway Analysis

To minimize variability in the protein dataset, protein intensity normalization was performed using NormalyzerDE [35]. The quantile normalization was applied to the relative expression after adding “1” to all values. Only proteins identified with an FDR ≤ 1% were included in the final protein list to construct high-confidence data. The impact of *V. amygdalina* leaf extract on signaling pathways in HeLa cells was analyzed using IPA. All proteins with significant alteration were input for core analysis to identify the related protein signaling pathways and upstream regulators with the detailed procedures and parameters for IPA analysis reported previously [36], and the comparison of all differential proteins against known canonical pathways in the IPA database (accessed on 29 February 2024) was performed. The activation and inhibition of protein pathways including the upstream regulators, the proteins influencing the expression of other proteins, were assessed based on all proteins with alteration and the adjusted *p*-value (z-score). The major signal transduction pathways were illustrated based on IPA results. Upstream regulators were considered significant if they had a z-score ≥ 1.5 and a *p*-value < 0.01. Biological pathway analysis was also conducted in the comparison of doxorubicin and the control group.

### 3.7. Imaging of Apoptotic Cells and Immuno-Based Early Apoptosis Protein Quantification

Live HeLa cells were treated with *V. amygdalina* leaf extract or doxorubicin for 24 h prior to the fluorescein (FITC) conjugated annexin V (Thermo Fischer, Waltham, MA, USA) and propidium iodide (PI) staining (Thermo Fischer, Waltham, MA, USA). Following the adjusted in-house method, the imaging of apoptotic cells was captured by using a fluorescence microscope (ZEISS Axio Observer with optical sectioning and deconvolution algorithm) using the green channel for annexin V at 495 and 519 nm as excitation and emission wavelengths, respectively. In addition, the dead cells stained by PI were taken in the red channel at 553 and 568 nm as excitation and emission wavelengths, respectively. 

The MILLIPLEX^®^ early apoptosis magnetic bead kit (Merck, Rahway, NJ, USA) was used to determine the levels of apoptotic protein markers in HeLa cells following the manufacturer’s instructions. Quantification of JNK, Bcl-2, Caspase-9, and p53 levels was performed using Luminex^®^ xMAP^®^ technology (Bio-Rad Laboratories, Hercules, CA, USA). After treating HeLa cells with *V. amygdalina* leaf extract at IC_50_ and negative control for 72 h, the cells were rinsed with ice-cold buffered saline and lysed with 0.3 mL of 1× MILLIPLEX^®^ Lysis Buffer (Merck, Rahway, NJ, USA) adding a protease inhibitor. The lysate was incubated at 50 °C for 10 min using vortex agitation to ensure complete lysis. Then the lysate was centrifuged at 14,000× *g* at 16 °C for 30 min to collect the supernatant. BCA protein assay was used to measure the concentration of proteins, and samples were adjusted to 2.5 μg μL^−1^ with PBS. Prior to the assay, the protein solution was further diluted in PBS to yield a final concentration of 0.5 μg μL^−1^. A 20 μL sample containing 10 μg of protein was used in the assay to quantify the level of apoptotic proteins and reported as median fluorescence intensity (MFI) values with a standard deviation of two biological replicates.

## 4. Conclusions

We have found that alkaloids, phenolic compounds, and steroids were the major secondary metabolites in *V. amygdalina* leaf extract. This composition of metabolites exhibited potential cytotoxic effects on cancerous HeLa cells at the IC_50_ value of 0.0767 ± 0.0334 µg mL^−1^, which was lower than that in the normal Vero cells at 4.043 ± 0.469 µg mL^−1^. The proteomics profile of HeLa cells treated with the extract revealed the proteins functioning in apoptosis and stress response were upregulated in a similar pattern to the group of doxorubicin, the anti-cancer treatment. The upstream regulator proteins with the most significant changes with *z*-scores ranging from −4.433 to +5.229, i.e., PTEN, XBP1, and ADORA2A, were involved in the regulation of apoptosis and cell death. Apoptosis was verified in HeLa cells treated with *V. amygdalina* leaf extract using annexin V/PI staining along with the immuno-based Luminex^®^ assay, which confirmed the significant increase in expression levels of apoptotic markers, JNK, p53, and caspase-9. This research uniquely highlighted the potential of *V. amygdalina* as a promising candidate for cancer therapy, offering valuable insights into its mechanism of action and laying the foundation for the future development of chemotherapy. However, further research on certain amounts of the composition of active compounds and their effects on specific biological targets is required for the development of an anti-cancer medication.

## Figures and Tables

**Table 1:** The most abundant 15 annotated compounds identified in the *V. amygdalina* leaf extract. The examples of unique fragment ions mass spectra (MS2) that elucidate the structure of nandrolene and deacetylvindoline are illustrated in the supplementary materials.

Name	Formula	Molecular Weight	Peak Area	Categories
Nandrolone decanoate	C_28_H_44_O_3_	428.3216	8.07 × 10^8^	Steroids
Deacetylvindoline	C_23_H_30_N_2_O_5_	414.2143	7.28 × 10^8^	Alkaloids
Rubiarbonone C	C_32_H_50_O_5_	536.3454	5.03 × 10^8^	Terpenes and terpenoids
Glycyrrhetinic acid	C_30_H_46_O_4_	492.3183	4.49 × 10^8^	Terpenes and terpenoids
Testosterone decanoate	C_29_H_46_O_3_	442.3378	2.23 × 10^8^	Steroids
Psoralidin	C_20_H_16_O_5_	336.1055	1.82 × 10^8^	Steroids
Ursolic acid	C_30_H_48_O_3_	456.3540	1.65 × 10^8^	Terpenes and terpenoids
3-Hydroxybupivicaine	C_18_H_28_N_2_O_2_	304.2111	1.64 × 10^8^	Alkaloids
Phenylglyoxylic acid	C_8_H_6_O_3_	150.0316	1.53 × 10^8^	Phenolic compounds
2-Hydroxyoctanoic acid	C_8_H_16_O_3_	182.0888	1.17 × 10^8^	Fatty acids and derivatives
Deidaclin	C_12_H_17_NO_6_	271.1056	7.48 × 10^7^	Glycosides
Prostaglandin F2	C_21_H_36_O_5_	368.2553	5.63 × 10^7^	Steroids
Licochalcone B	C_16_H_14_O_5_	308.0693	5.09 × 10^7^	Phenolic compounds
Prostaglandin E2-biotin	C_35_H_58_N_4_O_6_S	684.4016	3.75 × 10^7^	Steroids
Cafestol	C_20_H_28_O_3_	316.2089	2.44 × 10^7^	Terpenes and terpenoids

**Table 2:** The top ten remarkable upstream protein regulators that were predicted as activated (shown as positive *z*-score) or inhibited (shown as negative *z*-score) in HeLa cells after the treatment of *V. amygdalina* leaf extract.

Upstream Regulator	Molecule Type	*z*-Score	*p*-Value
HNF4A	Transcription regulator	2.67	2.44 × 10^−108^
LARP1	Translation regulator	5.229	1.34 × 10^−68^
MYCN	Transcription regulator	−3.682	1.76 × 10^−56^
NFE2L2	Transcription regulator	5.012	3.11 × 10^−41^
MLXIPL	Transcription regulator	−4.433	4.52 × 10^−37^
XBP1	Transcription regulator	3.704	3.06 × 10^−28^
STK11	Kinase	2.964	1.22 × 10^−27^
CLPP	Peptidase	−2.411	1.31 × 10^−27^
PTEN	Phosphatase	2.149	4.7 × 10^−16^
IL15	Cytokine	3.184	1.03 × 10^−14^

**Table 3:** Biological processes from GO annotation using 95 upstream regulators established six significant pathways involving apoptosis with ≤0.05 false discovery rate.

GO Term	Biological Process	False Discovery Rate
GO:0042981	Regulation of apoptotic process	0.00046
GO:0010941	Regulation of cell death	0.00073
GO:0010942	Positive regulation of cell death	0.0025
GO:0043068	Positive regulation of programed cell death	0.0037
GO:2001233	Regulation of apoptotic signaling pathway	0.0062
GO:0043065	Positive regulation of apoptotic process	0.0119

## Data Availability

Upon a reasonable request, the corresponding author is willing to provide the data and materials supporting the results of this study.

## Supplementary Materials

The following supporting information can be downloaded at: https://www.mdpi.com/article/10.3390/ph17081079/s1, Figure S1: The unique fragment ions spectrum (MS2) of nandrolene. Figure S2: The unique fragment ions spectrum (MS2) of deacetylvindoline.
