# Supplementary material for: Vernonia amygdalina Leaf Extract Induces Apoptosis in HeLa Cells: A Metabolomics and Proteomics Study
Source: Pharmaceuticals (Basel). 2024 Aug 16;17(8):1079. doi: 10.3390/ph17081079 (PMC11360076; doi:10.3390/ph17081079)
Supplement: Supplementary file 1 [file pharmaceuticals-17-01079-s001.zip › pharmaceuticals-3120926-supplementary.pdf]

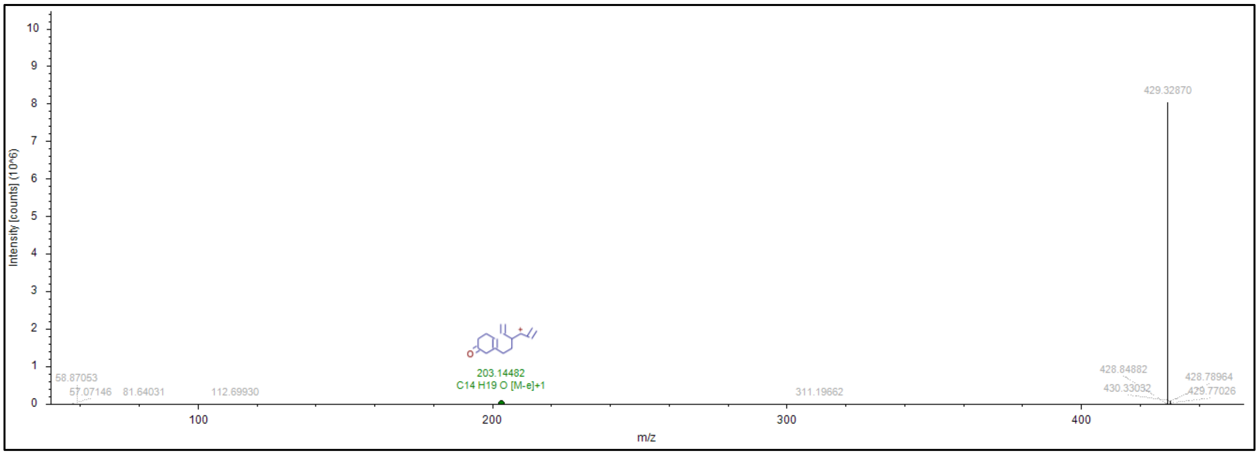

Figure S1: The unique fragment ions spectrum (MS2) of nandrolene.

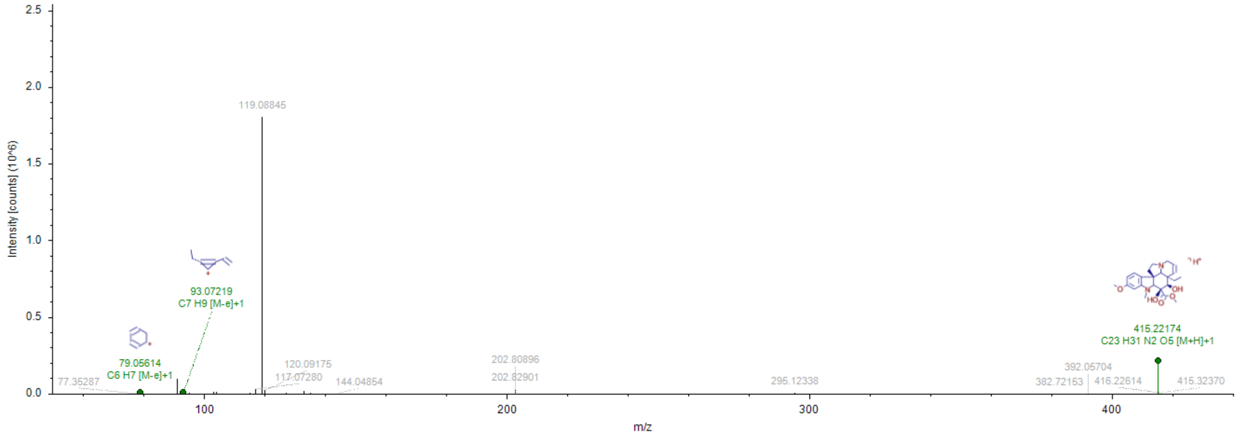

Figure S2: The unique fragment ions spectrum (MS2) of deacetylvindoline.
